# The Combined Effects of 6 Weeks of Jump Rope Interval Exercise and Dark Chocolate Consumption on Antioxidant Markers in Obese Adolescent Boys

**DOI:** 10.3390/antiox10111675

**Published:** 2021-10-24

**Authors:** Babak Hooshmand Moghadam, Reza Bagheri, Matin Ghanavati, Fatemeh Khodadadi, Neda Cheraghloo, Alexei Wong, Michael Nordvall, Katsuhiko Suzuki, Fatemeh Shabkhiz

**Affiliations:** 1 Department of Exercise Physiology, Ferdowsi University of Mashhad, Mashhad 9177948974, Iran; babak.hooshmand@mail.um.ac.ir (B.H.M.); Fateme.khodadadi@mail.um.ac.ir (F.K.); 2 Department of Exercise Physiology, University of Tehran, Tehran 1961733114, Iran; 3 Department of Exercise Physiology, University of Isfahan, Isfahan 8174673441, Iran; reza.bagheri@alumni.um.ac.ir; 4 National Nutrition and Food Technology Research Institute, Faculty of Nutrition Sciences and Food Technology, Shahid Beheshti University of Medical Sciences, Tehran 1416753955, Iran; matinghanavati@sbmu.ac.ir; 5 Department of Epidemiology and Biostatistics, School of Public Health, Tehran University of Medical Sciences, Tehran 1417613151, Iran; n-cheraghloo@razi.tums.ac.ir; 6 Department of Health and Human Performance, Marymount University, Arlington, VA 22207, USA; awong@marymount.edu (A.W.); mnordval@marymount.edu (M.N.); 7 Faculty of Sport Sciences, Waseda University, 2-579-15 Mikajima, Tokorozawa 359-1192, Japan

**Keywords:** antioxidants, dark chocolate, body composition, obesity, metabolic syndrome

## Abstract

Research has shown that both dark chocolate and exercise training may have favorable effects on antioxidant function in obese cohorts. However, their combined effect has not been established. We assessed the influences of six weeks of dark chocolate consumption combined with jump rope exercise on antioxidant markers in adolescent boys with obesity. Fifty adolescent boys with obesity (age = 15 ± 1 years) were randomly assigned into one of four groups; jump rope exercise + white chocolate consumption (JW; *n* = 13), jump rope exercise + dark chocolate consumption (JD; *n* = 13), dark chocolate consumption (DC; *n* = 12), or control (C; *n* = 12). Two participants dropped out of the study. Participants in JW and JD groups performed jump rope exercise three times per week for six weeks. Participants in the DC and JD groups consumed 30 g of dark chocolate containing 83% of cocoa during the same period. Serum concentrations of superoxide dismutase (SOD), total antioxidant capacity (TAC), glutathione peroxidase (GPx), and thiobarbituric acid reactive substances (TBARS) were evaluated prior to and after the interventions. All 3 intervention groups noted significant (*p* < 0.01) increases in serum concentrations of TAC, SOD, and GPx from baseline to post-test. In contrast, all intervention groups showed significantly reduced serum concentrations of TBARS from pre- to post-test (*p* ≤ 0.01). Bonferroni post hoc analysis revealed that post-test serum concentrations of TAC in the JD group were significantly greater than C (*p* < 0.001), DC (*p* = 0.010), and JW (*p* < 0.001) groups. In addition, post-test serum concentrations of SOD in the JD group were significantly greater than C group (*p* = 0.001). Post-test serum concentrations of GPx in the JD group were significantly greater than C (*p* < 0.001), DC (*p* = 0.021), and JW (*p* = 0.032) groups. The post-test serum concentrations of TBARS in the JD group was significantly lower than C (*p* < 0.001). No other significant between-group differences were observed. The current study provides evidence that dark chocolate consumption in combination with jump rope exercise is more efficient in improving antioxidant capacity than dark chocolate consumption or jump rope exercise alone among obese adolescent boys.

## 1. Introduction

The rising occurrence of obesity in adolescents has become a key public health concern [1,2,3]. Excessive accumulation of body fat characterizes this multifactorial condition and triggers a cascade of events that ultimately increases circulating free fatty acids (FFA) and deteriorates the metabolism of glucose [4,5]. Accumulation of energy substrates in the liver [6], muscle, and adipose tissues in obesity increases mitochondrial and peroxisomal oxidation, leading to a surge in free radical (FR) synthesis, oxidative stress, and damage to mitochondrial DNA [7]. Increased body fat also elevates concentrations of pro-inflammatory cytokines, including tumor necrosis factor-alpha (TNF-α), interleukin-1 (IL-1), and interleukin-6 (IL-6) by boosting reactive oxygen species (ROS) and nitrogen production in macrophages and monocytes [8,9,10,11]. Such chronic maladaptations were observed with obesity, resulting in reduced antioxidant capacity through decreased activity of antioxidative enzymes such as superoxide dismutase (SOD), catalase (CAT), and glutathione peroxidase (GPx) [12,13].

Various exercise interventions, including jump rope exercise [14], have been proposed as viable approaches to combat oxidative stress and improve antioxidant biomarkers in obese cohorts [15,16,17]. For instance, four 45-min jump rope exercise sessions per week for eight weeks improved SOD, GPx, and total antioxidant capacity (TAC) in young sedentary women [14]. Jump rope exercise has advantages of limited space requirements, equipment, and cost, consequently having great potential in boosting exercise adherence in obese adolescent cohorts. Moreover, evidence suggests that jump rope exercise improves various inflammatory and body composition indices in obese adolescents [18,19], which are subsequently important in regulating antioxidant function [20].

Dietary strategies are also often recommended to improve antioxidant markers [13] and capacity in obese populations [21]. The consumption of food-containing cocoa, such as dark chocolate, has gained significant attention in recent years due to beneficial antioxidant properties stemming from its high flavonoid content, including epicatechin, catechin, and proanthocyanidins [22,23]. Prior research investigating the effect of 4-week dark chocolate consumption showed improved DNA resistance to oxidative stress in healthy young individuals [24]. Moreover, 10 weeks of dietary cocoa consumption increased antioxidant response in obese mice with non-alcoholic fatty liver disease [25]. Research in healthy cohorts further indicates that combining exercise interventions with dark chocolate consumption enhances the antioxidant defense system. For instance, two weeks of cycling exercise combined with regular consumption of dark chocolate rich in cocoa polyphenols elicited decreased F2-isoprostanes (markers of oxidative stress) in active young males [26]. Additionally, acute pre-exercise dark chocolate consumption improved post-exercise plasma concentrations of antioxidant markers compared to exercise alone, and thus, decreased oxidative stress in healthy young men [27]. 

The beneficial effect of exercise on antioxidant performance in obese adolescents has been previously investigated [28,29]. However, the influence of chronic dark chocolate consumption alone and, perhaps more significantly, in combination with exercise remains unknown in this population. Therefore, we aimed to investigate the effects of dark chocolate consumption alone and in combination with exercise (jump rope exercise) on antioxidant markers (TAC, SOD, GPx, and thiobarbituric acid reactive substances (TBARS)) as secondary analyses/results of our prior work on inflammation and body composition in obese adolescent boys [18]. We hypothesized that jump rope exercise in combination with dark chocolate consumption would induce favorable changes on antioxidant markers.

## 2. Materials and Methods

### 2.1. Participants

Between January and March of 2019, 61 obese adolescent boys were screened for study participation. Of those, 50 met the criteria for baseline evaluation and were subsequently randomized to the JW, JD, DC, or C groups. After randomization, one participant in the JW and another in the JD group dropped out of the study for health and/or personal reasons. Forty-eight obese adolescent boys (age = 15 ± 1 years and height = 165.9 ± 4.9 cm) took part in the present investigation. Obesity was defined as a body mass index (BMI) ≥ 95th percentile for age and sex, according to guidelines for youth populations [30]. The inclusion criteria were as follows: ages 13 to 17 years, obese, body mass loss not exceeding two kilograms over the last six months, sedentary and/or contributing in less than 1 h of physical activity per week over the last year, and no recognized illnesses such as cardiovascular disease, diabetes, or similar. All participants were non-tobacco users and did not consume alcoholic beverages or caffeine at the time of data collection. Further exclusions were consumption of dietary supplements and medications potentially influencing muscle mass and fat metabolism, including amino acids, beta-blockers, beta-agonists, calcium channel blockers, and corticosteroids. The disease and health status information and all other exclusion criteria were attained by PAR-Q and evaluated by a physician [31]. Informed consent was obtained by participants and their parent/guardian before study enrollment. The study protocol (IR.SSRI.REC.1397.352) was approved by the Institute of Physical Education and Sports Sciences (Tehran, Iran) Human Subject Committee and undertaken in Mashhad, Iran. All experiments were carried out based on the Declaration of Helsinki. This study has been previously registered with the Iranian Registry of Clinical Trials (IRCT20151025024699N5). A priori sample size calculation was conducted using the G*Power analysis software (3.1.9.2). Based on prior investigations [14,32], α = 0.05, a power (1-β) of 0.80, and an ES = 0.4 (highest approximate effect size), a total sample size of at least 48 participants (*n* = 12 per group) was needed to establish statistically significant changes in several of our variables (TAC, SOD, GPx, and TBARS). An additional 10% (or 5 participants) were recruited for participation in case of attrition. 

### 2.2. Study Design

**Figure 1:**
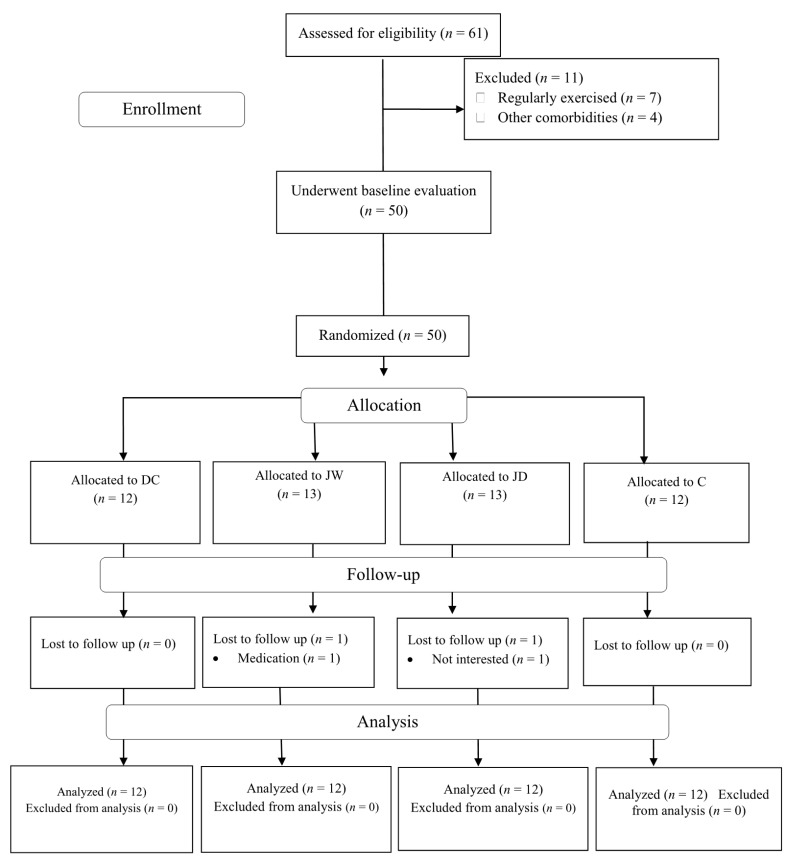
Participants flow diagram. Abbreviations: JW, jump rope exercise + white chocolate; JD, jump rope exercise + dark chocolate supplementation; DC, dark chocolate consumption; C, control.

The present investigation was performed between January and March of 2019. The study schematic design is shown in Figure 1. Prior to baseline assessments, participants were familiarized with all study procedures. Subsequently, participants were randomly assigned into one of four groups as follows: jump rope exercise + white chocolate consumption (JW; *n* = 13), jump rope exercise + dark chocolate consumption (JD; *n* = 13), dark chocolate consumption (DC; *n* = 12), or control (C; *n* = 12). Randomization assignment was stratified using commercially available software (available at www.randomizer.org, accessed on 1 September 2021). Assessments were performed at baseline and at six weeks post interventions (at minimum 48–72 h after the last jump rope exercise session). All assessments were performed concurrently (within 1 h) and under the same environmental conditions (~20 °C and ~55% humidity). Participants were directed to avoid altering their normal lifestyle and nutritional behaviors throughout the investigation. Participants in C group did not participate in any protocols and just maintained their normal lifestyle.

### 2.3. Anthropometry 

Participants were instructed to fast for 12 h (overnight fast, with at least 8 h of sleep) and abstain from physical activity and alcohol consumption for 48 h prior to data collection [33]. Participants’ body mass and height were measured with a digital scale (Lumbar, Hong Kong, China) to the nearest 0.1 kg and a stadiometer (Race Industrialization, Shanghai, China) to the nearest 0.1 cm, respectively. 

### 2.4. Blood Sampling and Laboratory Analysis

Fasting blood samples (20 mL) were taken from the median cubital vein using standard techniques following 12 h of overnight fasting. Blood samples were taken in all participants at baseline and 48 h after the final jump rope exercise session. Following blood sampling, samples were centrifuged at 3000 rpm for 10 min, and the serum was stored at −80 °C for future analysis of SOD (MyBioSource, San Diego, CA, USA; sensitivity: 1 ng/mL; intra assay CV: less than 8%; inter assay CV: less than 12%), TAC (MyBioSource; sensitivity: 0.14 U/mL; intra assay CV: less than 8%; inter assay CV: less than 10%), GPx (ZellBio, Lonsee, Germany; sensitivity: 5 U/mL; intra assay CV: 3.5%; inter assay CV: 4.7%), and malondialdehyde (MDA)-TBARS assay kit (Colorimetric) (ZellBio; sensitivity: 0.1 µM; intra assay CV: 5.8%; inter assay CV: 7.6%). To measure TBARS concentration, after preparing reagents, samples, and standards, we added 50 µL standards/samples to its related name test tubes. Then, 50 µL R4 regent was added and mixed. Moreover, 1mL Ready Chromogenic solution was added and consequently heated the mixture for one hour in boling water. Next, we cooled the test tubes in an ice bath and centrifuged them for 10 min at 3000–4000 rpm. We also pipetted 200 µL of pink color supernatant into the microplate and read the absorbance at 535nm. Finally, the concentration was calculated. 

### 2.5. Jump Rope Exercise Protocol

Jump rope exercise consisted of five sessions per week, 40 min/day for 6 weeks at specified times during the day (described below). Individual training sessions included 30 min of jump rope exercise with rest periods and warm-up and cool-down periods entailing 5 min of stretching for a total of 40 min. Jump rope exercise cadence was established via metronome and performed at a rate of 60 jumps/min for the 1st three weeks and proceeded to 90 jumps/min over the last 3 weeks. Individual bouts of jump rope exercise per training session and progressed over the 6-week study following short rest periods. Personal trainers supervised all jump rope exercise sessions who verified adherence to the exercise protocol. Our jump rope exercise protocol design is similar to prior investigations in obese populations [34], and is illustrated in Table 1. 

### 2.6. Dark Chocolate Consumption

Participants in both JD and DC groups consumed 30 g/d of dark chocolate containing 83% of cocoa (Farmand Gallardo 83 Percent Dark Chocolate) for six weeks. To reduce any psychological influence (placebo effect) of not being provided dark chocolate, participants in the JW group consumed white chocolate of the same shape and packaging (coated with aluminum foil) and devoid of cocoa [35]. Participants took their respective consumption daily as a midafternoon snack [36]. Table 2 shows the ingredients of 30 g of dark and white (placebo) chocolate utilized in this study. The dark chocolate dosage and timing were similar to a prior study showing the anti-inflammatory effects of dark chocolate [37]. To facilitate study compliance, participants were reminded weekly by phone call and WhatsApp software to consume the dark chocolate supplement. 

### 2.7. Nutrient Intake and Dietary Analysis

Intake and timing of nutrients (meals and snacks) in relationship to training sessions were controlled in the jump rope exercise group preceding and following training sessions. Participants consumed a banana 1 h prior to training that provided 0.30–0.35 g of carbohydrate per kilogram of body mass as a pre-training snack. Dinner was consumed 1.5–2 h after each exercise session and was standardized to contain 1.7 g/kg of carbohydrate, 0.3 g/kg of protein, and 0.4 g/kg of fat. These ratios were established based on recommendations by the Academy of Nutrition and Dietetics, Dietitians of Canada, and the American College of Sports Medicine for macronutrient distribution (55–65% of total calories from carbohydrates, ˂35% of total calories from fats, and 10–15% of total calories from protein).

It should be mentioned that all the above mentioned was standardized for every participant. Beyond this peri-training nutrient intake standardization, all participants were instructed to avoid altering their dietary habits during the study. Participants (with the help of their parent/guardian) were asked to submit 3-day (2 weekdays and 1 weekend) food records prior to and near the end of their respective assigned intervention [38]. Each food item was individually entered into the Diet Analysis Plus version 10 program (Cengage, Boston, MA, USA), and total energy consumption and the nutrient breakdown of proteins, fats, and carbohydrates were assessed and included for analysis.

### 2.8. Statistical Analyses

**Figure 2:**
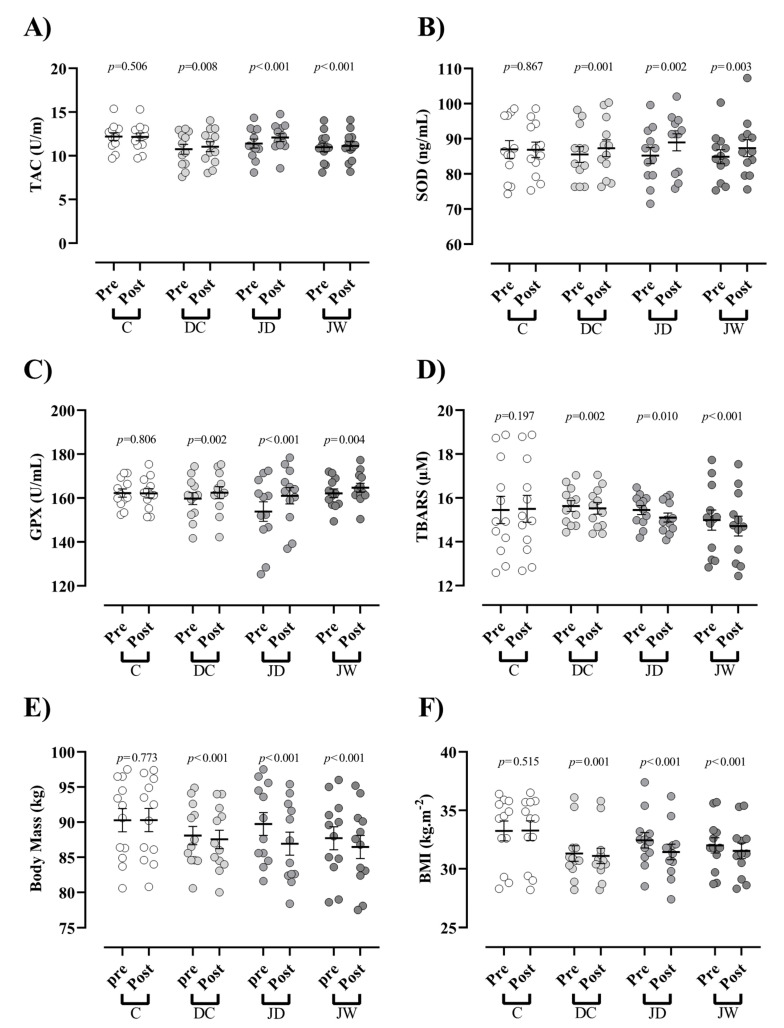
(**A**) Total antioxidant capacity (TAC); (**B**) superoxide dismutase (SOD); (**C**) glutathione peroxidase (GPx); (**D**) thiobarbituric acid reactive substances (TBARS); (**E**) body mass; (**F**) body mass index (BMI). **C**, control; **DC**, dark chocolate consumption; **JW**, jump rope exercise + white chocolate consumption; **JD**, jump rope exercise + dark chocolate consumption. Error bars indicate standard error of the mean (SEM).

Descriptive analyses were represented using mean ± standard deviation (SD). Analysis of variance (ANOVA) was used to compare the means of all between-group variables/results. Tukey’s honestly significant difference (HSD) test was used to determine whether the mean differences of variables/results were statistically different between two sets of groups. The Bonferroni test was further used to compare the mean values between each pair of groups. Figure 2 was generated using Graphpad Prism (version 8.4.3, San Diego, CA, USA). *p*-values less than 0.05 were regarded as statistically significant. Paired *t*-test were applied to compare the means of each variable in all groups at baseline and at the conclusion of the study. Analysis of covariance (ANCOVA) was used to evaluate whether the means of variables/results pre-and post-test were equal across. All analyses were performed in SPSS (version 26, Armonk, NY, USA).

## 3. Results

Compliance with jump rope exercise was nearly 92% for participants in both the JW and JD groups. Compliance with dark chocolate and white chocolate consumption was 100%. No adverse events were reported from dark chocolate consumption or jump rope exercise. In addition, no significant differences (*p* > 0.05) over time or between groups for mean daily energy intake and monitored protein, fat, and carbohydrate ratios consumption were observed. Results indicated no significant mean difference between groups for body mass and antioxidant markers at either baseline (Table 3) or post-test (*p* > 0.05). However, the mean of diff (post-test–pre-test) was significantly different in body mass and antioxidant markers between groups using ANOVA (*p* < 0.05). 

### 3.1. Antioxidants Markers

All three intervention groups noted significant increases in serum concentrations of TAC (DC = 0.3 U/mL (95% CI = 0.09 to 0.49; *p* = 0.008), JD = 0.69 U/mL (95% CI = 0.38 to 0.97; *p* < 0.001), and JW = 0.15 U/mL (95% CI = 0.08 to 0.22; *p* < 0.001); Figure 2A), SOD (DC = 1.78 ng/mL (95% CI = 0.88 to 2.67; *p* = 0.001), JD = 3.76 ng/mL (95% CI= 1.69 to 5.83; *p* = 0.002), and JW = 2.43 ng/mL (95% CI= 1 to 3.86; *p* = 0.003); Figure 2B), and GPx (DC = 2.71 U/mL (95% CI = 1.24 to 4.18; *p* = 0.002), JD = 7.2 U/mL (95% CI = 4.10 to 10.29; *p* < 0.001), and JW = 2.55 U/mL (95% CI = 1.01 to 4.08; *p* = 0.004); Figure 2C) from baseline to post-test. In contrast, all intervention groups showed significantly reduced serum concentrations of TBARS (DC = −0.11 µM (95% CI = −0.04 to −0.17; *p* = 0.002), JD = −0.34 µM (95% CI = −0.11 to −0.10; *p* = 0.010), and JW = −0.26 µM (95% CI = −0.19 to −0.34; *p* < 0.001); Figure 2D) from pre- to post-test.

### 3.2. Body Mass and BMI 

Body mass (DC = −0.5 kg (95% CI = −0.7 to −0.3, *p* < 0.001), JD = −2.8 kg (95% CI = −3.1 to −2 from baseline to post-test; *p* < 0.001), and JW = −1.2 kg (95% confidence interval (CI) = −1.5 to −0.9; *p* < 0.001); Figure 2E) and BMI (DC = −0.2 kg·m^−2^ (95% CI = −0.1 to −0.31; *p* = 0.001), JD = −1 kg·m^−2^ (95% CI = −0.88 to −1.1; *p* < 0.001), and JW = −0.4 kg·m^−2^ (95% CI = −0.34 to −0.59; *p* < 0.001); Figure 2F) were decreased in all three interventions.

### 3.3. ANCOVA

Bonferroni post hoc analysis revealed that post-test body mass and BMI decreased in the JD was significantly greater than other groups. Post-test serum concentrations of TAC in the JD group were significantly greater than C (*p* < 0.001), DC (*p* = 0.010), and JW (*p* < 0.001) groups. In addition, post-test serum concentrations of SOD in the JD group were significantly greater than C group (*p* = 0.001). Post-test serum concentrations of GPx in the JD group were significantly greater than C (*p* < 0.001), DC (*p* = 0.021), and JW (*p* = 0.032) groups. Lastly, post-test serum concentrations of TBARS in the JD group were significantly lower than C (*p* < 0.001). No other significant differences between groups were observed (Table 4).

## 4. Discussion

This study aimed to determine the effects of dark chocolate consumption alone and in combination with jump rope exercise on antioxidant biomarkers and capacity in obese adolescent boys. Of particular note, our study design indicated that; (1) all three intervention groups (JD, JW, and DC) demonstrated significantly increased post-test serum GPx, SOD, TAC, and decreased TBARS concentrations, (2) post-test alterations in GPx and TAC were greater in the JD group compared to the other groups, (3) SOD was significantly greater post-test in the JD vs. C group (no other group differences), and (4) reductions in post-test TBARS concentrations were significantly greater in the JD and JW groups compared to the C group (no other group differences noted). 

The trigger for obesity and associated comorbidities is intricately linked with a rise in ROS and subsequent oxidative stress [39]. This initiates a state of redox imbalance, where the pro-oxidants are excessively produced and the antioxidant defense mechanisms are weakened, simplifying a state of chronic inflammation [11,40]. Owing to the modulation of redox mechanisms in obesity, evidence suggests that a multimodal approach to treatment including diet changes, physical activity, and medical treatments may be successful in curbing oxidant stress [41,42]. Several investigations have indicated that habitual physical activity promotes an overall antioxidant response by eliciting a prerequisite low-grade inflammatory status [43,44,45]. Moreover, it has been suggested previously that increased plasma concentrations of antioxidant enzymes may occur in response to increased free radical generation during any exercise modality [46]. Nevertheless, among the several studies that have reported increased plasma concentrations of TBARS, SOD, GPx, and glutathione reductase (GR) and a decreased plasma α-tocopherol immediately following exercise, most have utilized aerobic or anaerobic-based exercises [46,47,48,49]. Fatouros et al. [44] demonstrated that three endurance-training sessions per week (walking/jogging at 50–82% of HR_max_ for 12–42 min, duration increased by 2 min every week) for 4 month negated exercise-induced lipid peroxidation and enhanced protection against oxidative stress by increasing TAC and GPx activity in an elderly population. Moreover, the effects of regular moderate to high-intensity exercise (incremental exercise test until exhaustion) on antioxidant status and oxidative damage biomarkers elicited a significant increase in SOD, GPx, and GR during recovery when compared to resting values in a younger population [43]. Further and with applicability to our results, four jump rope exercise sessions per week for eight weeks improved SOD, GPx, and TAC in young overweight and obese women [14]. This evidence along with our outcomes suggests the potential benefits of the improvement in antioxidant biomarkers, by means of jump rope exercise, in overweight and obese populations.

The enhanced effects of dark chocolate consumption combined with jump rope exercise (JD group) on the concentrations of TAC and GPx compared to the JW and DC groups may be attributed to several reasons. First, a primary result of the current trial showed that chocolate consumed five times a week in combination with jump rope exercise exerted greater body mass reductions compared to the JW and DC groups. Such greater body mass loss in the JD group may mask the promoting effects of dark chocolate consumption alone on antioxidant biomarkers as seen in the DC group. Even though the mechanisms underlying obesity-induced oxidative stress are not entirely clear, elevated leptin seen with obesity has been considered as a mechanism for regulating energy expenditure and mediating a pro-inflammatory state [50,51]. Interestingly, the increased circulating concentration of leptin has recently [39] been linked to higher pro-inflammatory cytokines such as TNF-α, a powerful activator of nicotinamide adenine dinucleotide phosphate (NADPH) oxidase, leading to the generation of ROS [52]. Insulin resistance seen in obese individuals may also have the effect of stimulating inducible NO synthase (iNOS) production, which in turn has been proposed to elevate peroxynitrite, a powerful oxidant [53]. Considering the role of obesity in underlying mechanisms mediating oxidative stress, body mass loss can independently negate obesity-induced oxidative stress and pro-inflammatory status [54,55,56]. Our current position toward the antioxidant effects of body mass loss are often predicated on research by Kelly et al. [57], who showed exercise training alone does not change the adipokine profile including C-reactive protein (CRP), TNF-α, IL-6, leptin, and adiponectin or oxidative stress in overweight children in the absence of body mass loss. A growing body of literature suggests the beneficial effects of exercise training and diet-induced body mass loss on anti-oxidative biomarkers among obese individuals [56,58,59,60]. Consistent with these notions are findings by Ghorbanian et al., who indicated body mass loss following rope training increased antioxidant defense and decreased oxidative damage and lipid peroxidation in sedentary females [14]. 

While it is not entirely clear whether exercise affects oxidative stress and inflammatory profiles in the absence of body mass loss, recent investigations have suggested the contrary. In this regard, Smjoo et al. [61] demonstrated that in the absence of body mass loss, three months of aerobic exercise training resulted in significant decreases in skeletal muscle-specific oxidative stress and systemic 8-isoprostane, as well as higher concentrations of mitochondrial antioxidants in untrained obese individuals. Further, following 6 months of aerobic exercise training, post-exercise elevated serum MDA decreased, and serum concentrations of SOD and GPx increased compared to exercise-induced responses pre-training [62]. However, despite using higher exercise intensities, exercise without body mass loss was unable to ameliorate markers of oxidative stress in obese adolescents during an 8-week exercise training period [57]. Despite these contrasting findings, it is generally accepted that low-grade oxidative status induced by various exercise modalities and subsequent mitochondrial stress significantly promotes mitochondrial-mediated adaptation and increases protection against oxidative damage [63,64]. Considering the diversity of exercise modalities and volume (intensity and duration), it should be carefully interpreted whether the adverse consequences of oxidative status/damage in the short-term resulting from acute bouts of exercise outweigh any potential benefits of chronic training on redox status. Our findings and prior research as indicated suggest quite the contrary, but a lack of well-designed protocols in healthy and/or recreationally active individuals investigating the effects of moderate intensity habitual exercise on oxidative stress biomarkers remains a present challenge. 

Another suggestion as seen from our results is dark chocolate consumption along with regular exercise training may have the effect of augmenting any alterations to biomarkers of oxidative stress than each intervention alone. As above, the discrepancy of results from studies conducted on induced oxidative damage following exercise has encouraged investigators to evaluate potential protective effects of various supplements touted to have antioxidants properties [64]. While several investigations promote the positive effects of antioxidants consumption on oxidative status [65,66,67], in contrast, high-dose antioxidant consumption, including vitamin C and E may induce adverse redox status effects through attenuation of exercise-induced signals that play a crucial role in post-exercise adaptations [68,69]. Therefore, attention has turned to dietary and naturally rich sources of antioxidant food sources such as coca and chocolate as opposed to conventional antioxidant supplements. Similar to our results, recent investigations have shown the advantages of short-term administration of coca polyphenols on endogenous antioxidant defense through analysis of enzymatic and non-enzymatic antioxidant activity. Both Cavarretta et al. [70] and Taub et al. [71] demonstrated beneficial effects of coca administration of at least 2 months on oxidative stress markers in football players and cyclists, respectively. Further, two weeks of dark chocolate consumption (80 g daily containing 197.4 mg polyphenols) and a double dose of cocoa polyphenols increased TAC concentrations on the testing day without negatively influencing pro-inflammatory myokines. Similarly, a significant reduction in F2-isoprostane concentration as a marker of lipoperoxidation by oxidative damage was seen in cyclists regularly ingesting dark chocolate [26]. It is worth mentioning that in addition to cocoa, other foods such as phenol-rich fruits have been shown to have antioxidant benefits during and following exercise through the identification of oxidative stress markers [72,73]. It has been proposed that the primary flavones found in cocoa seeds such as epicatechin, catechin, and procyanidins and its dopaminergic substances such as caffeine, theobromine, and theophylline act as potential natural antioxidants to attenuate exercise-induced endogenous oxidants and alter plasma markers of oxidative stress [74]. The functional hydroxyl group (OH) in polyphenolic compounds is thought to play a significant role in antioxidant defense by inhibiting ROS synthesis, chelating with trace elements responsible for ROS creation, scavenging excessive ROS production, and improving antioxidant defense [75]. These mechanisms of improved oxidative status following ingestion of phenol-rich foods alone and in combination with exercise continue to increase in attention and elucidate upon the complexity of responses seen in more robust research designs.

Concerning the significant reduction in TBARS concentration observed in the present study in the JD and JW groups (vs. control), such observed effects may be attributed to factors unrelated to dark chocolate consumption, including induced body mass loss and/or other mechanisms from regular exercise training alone causing lowered lipid peroxidation compared to baseline. This hypothesis has been raised before in that improvements in redox status may be a longer-term consequence of weight reduction and/or exercise training apart from acute exercise response [76,77,78]. It should be noted that participants in previous studies had higher plasma concentrations of baseline TBARS, perhaps a phenomenon of our particular participants characteristics. Further investigations in healthy individuals and other populations are needed to describe any synergistic effects of dark chocolate administration combined with habitual exercise training on TBARS concentrations. 

There are several limitations to this study. Although our jump rope exercise protocol was safe, practical, and analogous to previous research in obese adolescents [34], we did not control intensity using established methods such as the rating of perceived exertion or the percentage of heart rate or heart rate reserve; however, the American College of Sports Medicine does not indicate such monitoring as part of their current exercise recommendations for children and adolescents [79]. The JW and C groups in the present study were used to control different effects that could have influenced our results. Yet, it could be argued that the interpretation of our outcomes may not entirely account for certain unspecific effects of treatment (i.e., the placebo effect) and observation (i.e., the Hawthorne effect), which is also the case of most investigations in the health and nutrition fields. We measured four antioxidant markers in this study, which seems sufficient. Yet, measuring a higher number of antioxidant markers could have certainly strengthened our findings. Moreover, we did not assess blood glucose, lipid markers, or subcutaneous fat, which could have aided us in interpreting our outcomes. In addition, measuring baseline and post-intervention polyphenols would likely have provided a clearer picture of supplement adherence rates (JD, JW, and DC groups) or exercise differences (JD and JW groups) either within or between groups. Nevertheless, and to the best of our knowledge, this is the first study to investigate the influence of combining jump rope exercise with dark chocolate supplementation on oxidative status in obese adolescent boys. 

## 5. Conclusions

We conclude that dark chocolate supplementation in combination with jump rope exercise is more effective in improving antioxidant capacity than either dark chocolate supplementation or jump rope exercise alone in obese adolescent boys. It is possible that the body mass loss observed in participants and exercise-induced low-grade inflammation either independently or collectively are the primary underlying mechanisms responsible for our observed effects on antioxidant biomarkers and lipid peroxidation (TBARS concentration). Because over 90% of underage populations consume daily chocolate, and its consumption has been linked to positive health benefits [80,81], our findings may have important practical implications. Our results suggest that the beneficial effects of dark chocolate consumption in combination with jump rope exercise could be an encouraging factor for health-related outcomes in obese adolescent boys to combat obesity and subsequent oxidative stress. 

## Figures and Tables

**Table 1:** Jump rope exercise protocol for the JW and JD groups.

Week	Exercise			
	Sets	Duration per Set (min)	Rest Period (seconds)	Intensity (Jumps/min)
1	20	1 min	30 s	60
2	15	1.5	30 s	60
3	12	2	30 s	60
4	10	2.5	30 s	90
5	9	3	30 s	90
6	7	4	30 s	90

**Table 2:** Energy and nutrient composition of both dark and white chocolate supplements.

Content per Dose	Dark Chocolate	White Chocolate
Energy (kcal)	184.5	168.8
Total fat (g)	14.6	10.7
Carbohydrate (g)	5.1	14.7
Protein (g)	8.2	3.4
Cacao polyphenol (mg)	2650	0
Epicatechin (mg)	160	0
Caffeine (mg)	130	0
Theobromine (mg)	960	0
Flavonoids (mg)	450	0
Cocoa (%)	83	0

Abbreviations: kcal, kilocalorie; g, gram; mg, milligram.

**Table 3:** Descriptive characteristics of participants’ values represent mean standard error in brackets.

	C	DC	JD	JW
Height (cm)	164.3 (1.5)	167.8 (1.6)	166.2 (1.2)	165.5 (1.1)
Body mass (kg)	90.2 (1.6)	88 (1.2)	89.7 (1.6)	87.7 (1.6)
TAC (U/m)	12.1 (0.4)	10.7 (0.5)	11.3 (0.5)	10.9 (0.4)
SOD (ng/mL)	86.9 (2.5)	85.5 (2.2)	85.2 (2.3)	84.8 (2)
GPx (U/mL)	162.2 (1.8)	159.7 (2.7)	153.8 (4.4)	162.1 (1.9)
TBARS (µM)	15.4 (0.6)	15.6 (0.2)	15.4 (0.2)	14.9 (0.4)

**Abbreviations. TAC**, total antioxidant capacity; **SOD**, superoxide dismutase; **GPx**, glutathione peroxidase; **TBARS**, thiobarbituric acid reactive substances. **C**, control; **DC**, dark chocolate consumption; **JW**, jump rope exercise + white chocolate consumption; **JD**, jump rope exercise + dark chocolate consumption.

**Table 4:** The impact of group on the post values controlling pre values using ANCOVA and Bonferroni as multiple comparison test.

Dependent Variable	Contrast	β (SE)	95% CI	*p*-Value
Body mass-post	DC vs. C	−0.55 (0.18)	−0.04 to −1.05	0.026
	JD vs. C	−2.82 (0.18)	−3.32 to −2.32	<0.001
	JW vs. C	−1.25 (0.18)	−1.76 to −0.74	<0.001
	DC vs. JD	2.27 (0.18)	1.76 to 2.77	<0.001
	DC vs. JW	0.70 (0.18)	0.20 to 1.20	0.002
	JD vs. JW	−1.56 (0.18)	−2.0 to −1.0	<0.001
BMI-post	DC vs. C	−0.24 (0.07)	−0.44 to −0.40	0.010
	JD vs. C	−1 (0.07)	−1.22 to −0.83	<0.001
	JW vs. C	−0.5 (0.07)	−0.69 to −0.30	<0.001
	DC vs. JD	0.78 (0.07)	0.59 to 0.98	<0.001
	DC vs. JW	0.25 (0.07)	0.06 to 0.45	0.004
	JD vs. JW	−0.52 (0.07)	−0.72 to −0.33	<0.001
TAC-post	DC vs. C	0.27 (0.13)	−0.08 to 0.63	0.237
	JD vs. C	0.69 (0.13)	0.35 to 1.04	<0.001
	JW vs. C	0.14 (0.13)	−0.21 to 0.50	1.000
	DC vs. JD	−0.42 (0.12)	−0.76 to −0.08	0.010
	DC vs. JW	0.13 (0.12)	−0.21 to 0.47	1.000
	JD vs. JW	0.55 (0.12)	0.21 to 0.89	<0.001
SOD-post	DC vs. C	1.87 (0.95)	−0.76 to 4.50	0.334
	JD vs. C	3.85 (0.95)	1.22 to 6.48	0.001
	JW vs. C	2.52 (0.95)	−0.12 to 5.15	0.069
	DC vs. JD	−1.98 (0.95)	−4.61 to 0.64	0.256
	DC vs. JW	−0.65 (0.95)	−3.27 to 1.98	1.000
	JD vs. JW	1.34 (0.95)	−1.29 to 3.96	1.000
GPx-post	DC vs. C	2.58 (1.21)	−0.77 to 5.94	0.234
	JD vs. C	6.40 (1.26)	2.91 to 9.90	<0.001
	JW vs. C	2.69 (1.21)	−0.65 to 6.03	0.188
	DC vs. JD	−3.82 (1.24)	−7.23 to −0.40	0.021
	DC vs. JW	−0.11 (1.21)	−3.46 to 3.25	1.000
	JD vs. JW	3.71 (1.26)	0.22 to 7.20	0.032
TBARS-post	DC vs. C	−0.16 (0.09)	−0.41 to 0.09	0.506
	JD vs. C	−0.40 (0.09)	−0.64 to −0.15	<0.001
	JW vs. C	−0.33 (0.09)	−0.58 to −0.08	0.004
	DC vs. JD	0.24 (0.09)	−0.01 to 0.49	0.065
	DC vs. JW	0.17 (0.09)	−0.08 to 0.42	0.405
	JD vs. JW	−0.07 (0.09)	−0.32 to 0.18	1.000

**Abbreviations. BMI**, body mass index; **TAC**, total antioxidant capacity; **SOD**, superoxide dismutase; **GPx**, glutathione peroxidase; **TBARS**, thiobarbituric acid reactive substances. **C**, control; **DC**, dark chocolate consumption; **JW**, jump rope exercise + white chocolate consumption; **JD**, jump rope exercise + dark chocolate consumption.

## Data Availability

Data are available upon email to corresponding authors. The data are not publicly available due to faculty ethic roles.
